# Consumer Participation in Co-creation: An Enlightening Model of Causes and Effects Based on Ethical Values and Transcendent Motives

**DOI:** 10.3389/fpsyg.2016.00793

**Published:** 2016-05-26

**Authors:** Ricardo Martínez-Cañas, Pablo Ruiz-Palomino, Jorge Linuesa-Langreo, Juan J. Blázquez-Resino

**Affiliations:** Business Administration Department, University of Castilla-La ManchaCuenca, Spain

**Keywords:** value co-creation, ethical values, transcendent motives, information and communication technologies, ethical products, Marketing 3.0, conceptual paper

## Abstract

In the current highly interconnected modern world, the role of consumers has changed substantially due to their active collaboration with companies in product and process innovation. Specifically, consumer participation has become key to the development of successful products and services, as companies have come to rely more and more on consumers' opinion as a source of innovative ideas and brand value. However, whereas existing research has focused on identifying the different elements involved in consumers' co-creation, there is still the need to comprehend better this complex mechanism by integrating distinct dimensional insights. With an integrative review of research into three important perspectives, one nurturing from *the Service-Dominant logic*, another one based on *the information and communication technologies (ICTs) platforms, and (the ethical values-driven) Marketing 3.0 paradigm*, this article proposes a conceptual framework in which consumers' *ethical values and transcendent motivations* play an important role in encouraging their engagement in co-creation activities. In this connection, and with consumers increasingly embracing the need to fulfill a social and ethical function in society, the co-creation process is here comprehended as a means to emphasize the social and moral aspects of co-creation. This article also identifies the important, supportive role of the Marketing 3.0 paradigm and Web 3.0 tools to initiate the co-creation process, as well as the important valuable benefits attained by both companies and consumers after consumers engage in this process. Importantly, these benefits are highlighted to increase when ethical products are the object of these co-creation activities. All these insights have notable implications for both research and managerial practice.

## Introduction

In the recent times value co-creation has emerged as a major strength of companies to remain and gain competitiveness (Zwass, 2010). Defined as a holistic management strategy focused on bringing distinct agents together to produce mutually valued outcomes (Prahalad and Ramaswamy, 2004), this is increasingly utilized by companies as a source of corporate reputation, brand value, and competitive advantage (Cova and Dalli, 2009). Beyond the conception of market as company centric-based, where economic exchange is about making and distributing things to be sold (Good-Dominant Logic, see Vargo and Lusch, 2004, 2008), companies are increasingly conceiving markets as the intersection of companies, network partners, and consumers to co-create value (Service-Dominant Logic, see Vargo and Lusch, 2008; Vargo et al., 2008; Williams and Aitken, 2011). This new understanding has guided in practice to conceive consumers, along with their personalized experiences, not as passive but rather active players to create value in designing and developing products/services (Prahalad, 2004; Prahalad and Ramaswamy, 2004).

While co-creation research has importantly advanced our understanding around the concept in the last decade (Prahalad and Ramaswamy, 2004; Holbrook, 2006; Grönroos, 2008; Payne et al., 2008; Vargo and Lusch, 2008; Zwass, 2010; Brodie et al., 2013), relatively little knowledge exists about how consumers engage in co-creation. Whereas, there have been some major attempts to understand this process (see Payne et al., 2008; Brodie et al., 2013), the understanding of the concept is still far complete. Our review of literature notes that interesting pieces of this complex puzzle are still missing (see Arvidsson, 2011), and an integration of the different existing perspectives around the concept, which has not yet been well addressed in literature (see Edvardsson et al., 2011), appears to be essential.

One important hitherto area of research has involved studies around the co-creation processes nurturing from the Service-Dominant logic (S-D logic), which posits consumers as resource integrators, value creating entities as traditional companies are (Vargo and Lusch, 2008; Edvardsson et al., 2011). With this theory in mind, previous research has studied consumers' involvement in co-creation to understand elements involved for the process to occur (Cova and Dalli, 2009; Brodie et al., 2013; Roberts et al., 2014). One of these important elements refers to motivations driving consumers to participate in co-creation activities. Specifically, and from a psychological perspective, studies have revolved around either intrinsic or extrinsic motives to explain why consumers participate in value co-creation processes (Roberts et al., 2014). In addition, under the umbrella of this (S-D logic) perspective, another important stream of research has focused on examining and identifying the positive outcomes deriving from these processes (i.e., consumer satisfaction, consumer learning, consumer brand loyalty; Payne et al., 2008; Bowden, 2009; Jaakkola and Alexander, 2014; Luo et al., 2015).

One another perspective encompasses an array of studies (see Kalaignanam and Varadarajan, 2006; Zwass, 2010; Garrigos-Simon et al., 2012) stressing the significant influence of *information and communication technologies (ICTs) platforms* to initiate and develop this process. These studies have much emphasized how the rapid diffusion of the *ICTs* have facilitated both social interaction and virtual communities, which notably enhances productive co-creation processes. Indeed, important elements that have facilitated consumers' access to information and social relationships include the unstoppable spread of the Internet, the *ICTs*, the Web 2.0 (social networks), consumer relationship management, and even the more recent Web 3.0 platforms, which offer improved interaction and learning through data mining, semantic webs, and artificial intelligence. These technologies and tools support a better understanding of consumers' existing and potential needs and problems. In particular, Web 3.0—with the support of social media tools and intelligent technologies—enables bidirectional conversations between consumers and companies, and the Web 3.0 technology platforms are designed to engage consumers in a collaborative interaction that provides mutually beneficial value (Choudhury and Harrigan, 2014). Interactions thus, are part of the modern, global era, which are greatly favored by *ICTs*, thus, in turn, contributing to foster consumers' active and valuable collaboration with companies in product and process innovation.

Although both perspectives are critically formative of the existing body of research, in the last times some studies (see Payne et al., 2008; Edvardsson et al., 2011) have discussed the need to introduce new theories to better understand the consumer's co-creation process. In this connection, recent literature has begun to nurture from a new, very incipient perspective (i.e., Kotler et al., 2010; Arvidsson, 2011; Williams and Aitken, 2011), *the ethical values-driven Marketing 3.0 paradigm* (Kotler et al., 2010), to investigate this process. This new understanding turns around the idea that consumers are increasingly seeking solutions to their own concerns and are interested in building a better world, guided by their ethical values in purchasing decision processes (e.g., Hollenbeck and Zinkhan, 2010). It understands, as observed in several studies (i.e., Shaw et al., 2005), that consumers, prior to show affinity to brands, attend to the benevolence, security, equality, and environmental responsibility that brands and products exhibit. In other words, consumers' choices of products and services are increasingly based on the extent to which they permit to fulfill their higher-order needs for social, economic, and environmental justice (Kotler et al., 2010). This applied to our research subject would entail other type of motives, beyond those of an intrinsic or extrinsic nature, for consumers to involve in co-creation activities, namely, transcendent motives (e.g., Pérez-López, 1998; Guillén et al., 2014). Including thus transcendent motives in our understanding of the issue might fill a research gap that exists in the current knowledge about how and why consumers engage in value co-creation processes.

Given the attractive this new perspective might offer to gain better understanding of consumers' co-creation processes, this study tries to utilize and integrate it together with the other aforementioned areas of research. Specifically, and following examples of Payne et al. (2008) and Edvardsson et al. (2011), this article seeks to do an integrative literature review around these three important perspectives, *the S-D logic*-based area of research, *the ICTs platforms*-based understanding, *and the ethical values-driven Marketing 3.0 paradigm*. Thus, this article tries to shed light on several fundamental questions:

- What are the antecedents (i.e., extrinsic, intrinsic, or ethical motivators) of consumers' engagement in co-creation processes?, or in other words, why do consumers engage in value co-creation activities with companies?
- What roles do the Marketing 3.0 paradigm, Web 3.0 platforms, and *ICTs* serve in value co-creation processes?
- What is the process by which co-creation leads to positive outcomes?
- What are the outcomes of co-creation for both consumers and companies?

In seeking answers to these questions, our conceptual approach and subsequent model tries to develop a better understanding of both antecedents and consequences of consumers' engagement in co-creation activities. Importantly, our study introduces missing elements in this area, very related to ethics and transcendent motives, which help form a substantial source of information around the principal elements involved in consumers' co-creation processes. It should be of assistance to both scholars and practitioners who seek to understand or optimally manage consumers' engagement in co-creation activities.

After this introduction, our second section describes the method (i.e., integrative literature review) we used to gain a new enlightening perspective on the consumers' co-creation process. The third section puts forward the conceptual analysis we have undertaken regarding the different elements involved in understanding consumers' engagement in co-creation activities, which produces a series of theoretical propositions. Next, a fourth section synthesizes and links all these elements in a form of conceptual model that integrates both causes and effects of the co-creation process. In the final sections, the article outlines a series of conclusions and offers compelling implications for both research and practice. Limitations of our study, and avenues for future research are also finally outlined.

## Methods

In this paper an integrative literature review has been conducted as a distinctive form of research to generate new knowledge to literature (Torraco, 2005; Yorks, 2008). Specifically, our integrative literature review aims to elucidate good understanding around the important elements involved in consumers' intentions to co-create with companies. This method has emerged in recent years as a relevant one to criticize and synthesize various streams of literature (Shuck, 2011), and importantly, to set up new theoretical frameworks and research agendas in the social sciences field (i.e., Carasco-Saul et al., 2015; Mercurio, 2015).

Although an integrative literature review can be also used to investigate mature topics and emergent topics, the latter ones, which are relatively new and lack a comprehensive state of art, can benefit importantly from holistic conceptualizations and literature syntheses. In this paper we address an emerging topic in the marketing and consumer behavior literature, so accordingly, this method could help substantially to bring into light new perspectives (i.e., ethical values, Marketing 3.0, transcendent motives) in our understanding on both antecedents and positive effects of consumers' engagement in co-creation activities. Thus, thanks to using this research method, a value-added theoretical contribution widening the classical economical perspective of the value co-creation phenomenon, has been feasible.

Following the criteria used in this method to conduct this type of research proficiently (Torraco, 2005; Yorks, 2008), the most important bibliographical databases were primarily selected. Research databases such as Web of Science, Scopus, PsycoInfo, ABI/Inform, JSTOR, the Academy of Management database, EBSCO Academic Search Premier, and Google Scholar were utilized to access the literature. Next, these databases were used to conduct an electronic search for published articles around our subject matter of this study, through including primary keywords. With a coverage of databases during a 15 years period (2000–2015), search words included “value co-creation” AND (business OR management OR psychology OR organizations OR motivation OR ethics OR transcendent OR marketing OR Web 3.0). In addition, criteria used for retaining or discarding articles was threefold: relevance of the title, diffusion relevance (times cited), and inclusion in the abstract of terms such as “motivation” “transcendent” “value co-creation” “marketing” “Web 3.0” and “ethics.” Also, a deep inclusion-exclusion criteria was used after a complete reading of each pre-selected article. Finally, due to their important scholarly status, other relevant references were included.

All these articles were selected to analyze the existing research under the perspective of elucidating links between the different elements involved in the consumers' value co-creation phenomenon. Thus, the selected papers for our integrative review show their high relevance to better understand our topic of interest. Specifically, they allowed us to widen the existing current perspective around the theme by including the important role of ethical values, Marketing 3.0 and transcendent motivations in driving consumers' engagement in value co-creation activities. Thanks to this theoretical integration our article represents valuable research in conceptual terms, providing direction for researchers and practitioners who are interested in studying or developing, leveraging, and managing, value co-creation activities.

## Analysis and propositions

### Co-creation and consumer 3.0

#### Value co-creation

The value co-creation concept often refers to company mechanisms, such as co-design (Lusch et al., 2007), co-production (Auh et al., 2007; Sanders and Stappers, 2008), consumer participation (Bendapudi and Leone, 2003; Bagozzi and Dholakia, 2006; Fang et al., 2008; Chan et al., 2010; Olsen and Mai, 2013), user innovation (Barki and Harwick, 1994; Lagrosen, 2005), customer engagement (Bowden, 2009; Jaakkola and Alexander, 2014), pro-consumption (Richards, 2011), or co-innovation (Lee et al., 2012). In essence, value co-creation ultimately is a holistic management initiative or economic strategy that brings different parties together to produce a mutually valued outcome (Prahalad and Ramaswamy, 2004).

Research into the value co-creation concept has importantly configured and evolved thanks to the emergence of the S-D logic, which brings to light the important ingredient consumers (and other agents) represent in product and process innovation (see Vargo and Lusch, 2008; Vargo et al., 2008; Williams and Aitken, 2011). However, based on this S-D logic new understanding of creating value though, various other theoretical approaches have been emerging around the concept (Saarijärvi et al., 2013), including the service science, service logic, many-to-many marketing, social constructionist, new product development, or post-modernism perspectives. Table 1 summarizes these key approaches, ideas, concepts, and authors, and shows they differ, to some extent, in their characteristics and *locus* of attention (e.g., companies, customers, communities, networks). Consequently, value co-creation as a concept lacks a clearly united basis for further development. Yet this divergence also provides an interesting starting point for addressing important questions about who benefits from the created value, what kind of resources are used, and what mechanism (or technology) defines how company resources get integrated into customer processes (Saarijärvi et al., 2013).

**Table 1:** **Theoretical approaches to value co-creation concept**.

**Theoretical approach**	**Main ideas and contributions**	**Key authors**
Service dominant (S-D) Logic	Service, not goods, is the fundamental unit of exchange. Co-created value becomes a joint function of actions by the provider(s) and consumer(s). For services to be delivered, consumers must learn to use, maintain, repair, and adapt offerings to their unique needs, usage situations, and behaviors.	Vargo and Lusch, 2004, 2008
Service science	Based on the S-D logic, service science analyzes value co-creation as configurations of people, technology, and value propositions. It integrates existing resources with those available from a variety of service systems that can contribute to system well-being, as determined by the system's environmental context.	Spohrer et al., 2008; Baron and Harris, 2010
Service logic	It is not the customer who becomes a value co-creator with a supplier; rather, it is the supplier that adopts its service logics and develops firm–customer interactions as part of its market offerings, such that it can become a co-creator of value with customers. Interactions ensure that value-in-use equates with the value proposition.	Grönroos, 2008, 2011
Many-to-many marketing	Customer networks have a key role (not dyadic firm–customer relationships) in value co-creation. Relations include a multitude of actors—intermediaries, employees, actors, and society in general—and generate value co-creation.	Gummesson, 2007, 2008
Social constructionist	Value co-creation is located within a social context; that is, it is value-in-social-context (not value-in-use), a view that captures the holistic nature of value.	Edvardsson et al., 2011
New product development	Following the more active role of customers, firms increasingly engage customers in new product/service development processes. New customer roles include product conceptualization, design, testing, support specialization, and product marketing. Customers are proactive.	Nambisan and Nambisan, 2008; O'Hern and Rindfleisch, 2010
Post-modernism	Firms shift toward offering more tailored goods and services to consumers to allow their active participation, such that they must open up more of their processes.	Firat and Venkatesh, 1993; Bendapudi and Leone, 2003

Analyzing the main differences among these theoretical approaches in detail reveals that value co-creation is based on interactive processes, promoted by agents with valuable resources that they could offer up for integration (Prahalad and Ramaswamy, 2004). In addition, value co-creation emphasizes joint efforts by companies, consumers, and other agents, such that reciprocity and mutual dependence are particularly important in defining the interdependent roles associated with the production of services and value creation (Vargo et al., 2008). In the participation among agents, this co-created value arises in the form of personalized, unique experiences derived from the value-in-use for the customer or value-in-context in general. These benefits, together with ongoing revenues, learning, and enhanced market performance, can drive some desired effects for both companies (e.g., trust, commitment, loyalty, risk reduction, cost effectiveness) and consumers (e.g., empowerment, commitment, satisfaction, learning, personalized experiences).

According to a classical value creation approach, companies offer innovative products (Kirca et al., 2005) by leveraging their distinctive, differentiated capabilities to create great value for consumers and achieve competitive advantages. In the value co-creation paradigm, companies instead co-create such benefits together with consumers (or other agents), with a more humanistic view, which ultimately might enhance consumers' loyalty, based on their own perceptions (Slater and Narver, 1995; Füller, 2006). Furthermore, consumers must be willing and able to interact with companies and contribute to the process, which constitutes a key challenge (Lengnick-Hall et al., 2000; Sawhney and Prandelli, 2000; Auh et al., 2007; Füller and Matzler, 2008). Understanding consumers is not enough to ensure new product success; consumers also must be active or proactive (Lagrosen, 2005), as well as intrinsically or extrinsically motivated to share their knowledge, ideas, and preferences with companies (Füller, 2006). For example, consumers' *leit motive* could relate to activities that lead to unique experiences, which then would involve both customer participation and a connection to the experience (Shaw et al., 2011). Ensuring the success of a new product or service thus requires (among other factors) a more humanistic, detailed understanding of consumers' ethical values and transcendent motives, which determine their behavior. But acknowledgment of the concrete exchange situation (product-service characteristics, technological platform) also is critical. Therefore, using a marketing strategy that is oriented toward social aspects and defining the appropriate role of *ICTs* and Web 3.0 platforms represent important elements.

#### Marketing 3.0, web 3.0, and ICTs platforms

Marketing 3.0 represents the most recent marketing paradigm, with the key assumption that companies treat consumers as human beings with intelligence, heart, soul, and spirit (Kotler et al., 2010). As such, it is a prominent philosophy, gaining relevance among consumers who increasingly recognize the effects of unpredictable social, economic, and environmental changes on them (Kotler et al., 2010). Previous paradigms included Marketing 1.0, which emerged during the industrial, “product-centric” era and focused on mass sales of products based on functional value propositions; the marketing department's activities centered solely on the product or service for sale. An enhanced version, Marketing 2.0, arose during the information age, such that companies adopted an emotional value proposition. That is, Marketing 2.0 is based not on product transactions but on the relationships that allow companies to engage consumers with messages and individualized services and products (Corbae et al., 2003). Consumers differ in their preferences, so companies must segment the market and develop unique products for different consumers (Kotler et al., 2010).

The new paradigm of Marketing 3.0 implies that we are at the dawn of a “values-driven” era, characterized by populations who want to satisfy functional, emotional, and spiritual needs through their consumption. Marketing 3.0 seeks to satisfy the whole person (mind, body, soul). This evolution to more human-centric value propositions is shaping the future of marketing in three main ways (Kotler et al., 2010). First, mass participation and co-creation through collaborative marketing reflect how modern social media and the Internet have tapped into natural human desires for connectivity and interactivity. Companies thus seek collaborative marketing strategies, such as product or service co-creation, with consumers, employees, channel partners, and other companies that have similar goals and values. Second, in the globalization paradox, technological advances have created truly “global citizens” who still want to be considered individuals. Accordingly, marketing needs to address both local and global communities simultaneously. Third, the rise of a creative society and human–spirit marketing encourages creative people, who tend to innovate, collaborate, and express themselves more than others, to pursue their self-actualization but also demand originality and trendiness in the products and services they consume.

Therefore, the conceptual approach of Marketing 3.0 entails a redefined triangle: Rather than brand, positioning, and differentiation, it builds on this formula to suggest a 3-I model, encompassing *identity, image*, and *integrity* (Kotler et al., 2010). The first I, *identity*, reflects the relationship between positioning and brand and seeks to address the rational portion of the value proposition. *Image* instead lies at the juncture of differentiation and brand and strives to capture the emotions of the target audience. Finally, *integrity* represents the intersection of positioning and differentiation, and it aims to fulfill the brand promise authentically while fostering trust, commitment, and loyalty. This 3-I model demonstrates how more intangible and social factors can determine the real and perceived value created by a company.

Accordingly, we anticipate that, in line with the Marketing 3.0 paradigm, value co-creation is an integrated process of proactive, creative, and social cooperation among companies and consumers. Through various value co-creation activities, organizations attract consumers, and engage them in discussions of their emotions, feelings, and expectations, thereby generating a constructive, deep exchange of ideas, resources, and services (Piller et al., 2010). Furthermore, these exchanges are more plausible as a result of the new advances in *ICTs*, including Web 3.0 platforms. These platforms represent a third generation of Internet-based services that collectively comprise what might be called “the intelligent Web,” which includes semantic webs, micro-formats, natural language searches, data mining, machine learning, recommendation agents, and artificial intelligence technologies (Markoff, 2006). These services explicitly emphasize machine-facilitated understanding of information as a means to provide a more productive, intuitive user experience. Therefore, Web 3.0 enables customers to converge with companies through several emerging technology trends, such as ubiquitous connectivity, network computing, open technologies, open identity, and a more intelligent web.

From another perspective, Web 3.0 and other *ICTs* platforms constitute the technical elements needed to implement Marketing 3.0 strategies, because they enable consumers to create value by facilitating their collaborations with companies (Kalaignanam and Varadarajan, 2006) while also increasing the adaptation and personalization of products, brands, and services by and for different users, according to their own needs (Garrigos-Simon et al., 2012). Such technological advances offer new tools that consumers can use to interact, as well as incentives for creating new products and services. The ubiquity of the Internet, Web 3.0, and *ICTs* also allows users to interact widely and easily, with both companies and other users. The Internet has increased consumers' power, through two main processes: reformulating the identity of each user (through interactions with others, learning processes, and the creation of social links) and increasing users' efficiency and skills (Amichai-Hamburger et al., 2008). These tools also have an important role in helping companies gain advantages for the design and delivery of customized products that maximize consumers' satisfaction (Du et al., 2006). However, more skilled and powerful consumers need *ICTs* to help them proactively generate and evaluate new ideas, improve product details, select and personalize preferred prototypes, experience new product features (e.g., through simulations), obtain and share new product information, and participate in the development of new products (Füller, 2006). Thus, we propose:

Proposition 1: Web 3.0 platforms and the generalization of new and advanced *ICTs* boost consumers' engagement in value co-creation activities.

### Consumers' co-creation motives: Ethics and transcendence

#### Consumers' motivations to co-create

Motivation is an antecedent of human behavior, explaining why people behave in certain ways, what provokes these behaviors, and what directs subsequent voluntary actions (Deci and Ryan, 1985; Nambisan, 2002). Prior literature explicates what motivates people to act, using various theories that attempt to detail the entire human motivation process (Ambrose and Kulik, 1999). Relying on seminal work on human motivations by Guillén et al. (2014), we offer a simplification and integrative review, with the goal of providing theoretical support for the motivation process involved in co-creation. Thus, we present two basic motivational approaches: Maslow's (1943) and Herzberg's (1968).

Maslow's *Theory of Human Motivation* (1943) classifies motivations according to whether they seek to meet basic, lower-order, physiological needs (food, water, safety, and security) or higher-order needs linked to social activities, such as esteem-building, self-actualization, or continuous self-improvement. These needs act as motivators until they are satisfied, though some exceptions are possible (Maslow, 1943). This theory is based on two essential pillars: Human needs follow a hierarchical pattern, and there is a dynamic between them. Thus, the motivation to satisfy a higher-order need should exist only if lower-order needs already have been satisfied.

Expansions of Maslow's framework generally propose similar classification patterns. For example, Herzberg et al. (1959) rely on Maslow's description of the hierarchy of needs and divide motivations into *hygiene factors* (i.e., company policy, relationship with peers, or security) and *motivator factors* (i.e., achievement, recognition, responsibility, advancement). For these authors, only the latter are true motivators, because the hygiene factors actually cause de-motivation if people lack them, whereas their presence does not exert an effect in motivational terms. McClelland (1962) also identifies three types of needs (achievement, power, and affiliation) that prompt three associated motivations. Finally, Alderfer's study (Alderfer, 1969) extends Maslow's theory by categorizing human needs into three types: *existence*, which comprises Maslow's basic needs; *relatedness*, encompassing Maslow's social and external self-esteem needs; and *growth*, which connects closely with Maslow's internal self-esteem and self-actualization needs. All in all, these theories provide a greater general understanding of human motivations and needs, for both managerial research and practice.

Another important description of human motivations comes from Herzberg (1968), who distinguishes extrinsic from intrinsic motivational factors. When people are intrinsically motivated, they experience interest and enjoyment, feel competent and self-determining, and hold an internal locus of control, such that they perceive themselves as the masters of their destinies and outcomes, through their behavior. Conversely, when people are extrinsically motivated, they need external factors such as money or verbal support to motivate them to act. Thus, intrinsic factors are inherent to the person/actor, whereas extrinsic factors are facilitated outside of the person/actor (Heath, 1999).

Again, others have developed and enriched Herzberg's (1968) intrinsic–extrinsic theory with subsequent research. For example, self-determination theory (Deci and Ryan, 1985; Ryan and Deci, 2000) relies heavily on the concepts of intrinsic and extrinsic motivation, such that *competence* (to succeed in difficult tasks and be able to achieve expected results) and *autonomy* (to have the ability to choose) needs are described as intimately related with intrinsic motivations, whereas *relatedness* (to establish a sense of mutual respect and trust with others) is classified as an extrinsic type of motivation.

As summarized in Table 2, consumers then might be encouraged to participate in co-creation activities to attain *financial rewards* or acquire *useful skills for career advancement* purposes, as well as *personal relationships* and *social capital* resources that help *construct their own identity* (e.g., Zwass, 2010; Roberts et al., 2014). Consumers might also participate if they believe doing so facilitates their *access to social standing and reputation* (Zwass, 2010; Chen et al., 2012). From this activity, they can *learn* what they appreciate most (Wasko and Faraj, 2000; Zwass, 2010), and in many cases, participation appears valuable in itself, enabling them to meet their *self-esteem, self-efficacy*, and *self-expression* needs (Bandura, 1995; Kollock, 1999). Furthermore, their interests might be motivated *altruistically* (Kollock, 1999; Roberts et al., 2014) or because of *feeling joy* and *enjoyment* (i.e., *hedonic motivations*) by doing what they love (Nambisan and Baron, 2009; Roberts et al., 2014). In general, our literature review thus elucidates various different needs that lead consumers to co-create, which can clearly be grouped into the higher–lower or intrinsic–extrinsic motivational taxonomies (see Table 3).

**Table 2:** **Consumers' motives for participating in co-creation processes**.

**Motivational factors**	**Authors**
*Financial rewards*—indirect and direct monetary payoffs from co-creation activities.	Wasko and Faraj, 2000; Füller, 2006; Hoyer et al., 2010; Zwass, 2010; Roberts et al., 2014
*Career advancement*—acquiring skills and experience, becoming known	Lerner and Tirole, 2002; Zwass, 2010; Roberts et al., 2014
*Acquiring social capital, personal relationships, and identity construction—*co-creators derive a sense of identity from co-creating communities and projects	Wasko and Faraj, 2000; Nambisan, 2002; Füller, 2006; Nambisan and Baron, 2009; Hoyer et al., 2010, Zwass, 2010; Chen et al., 2012; Roberts et al., 2014
Access for *social standing* and *renown*	Lerner and Tirole, 2002; Nambisan, 2002; Füller, 2006; Nambisan and Baron, 2009; Hoyer et al., 2010; Zwass, 2010; Chen et al., 2012; Roberts et al., 2014
*Self-esteem, self-efficacy*, and *self-expression*	Bandura, 1995; Kollock, 1999
*Learning* through co-creation from and with others	Wasko and Faraj, 2000; Zwass, 2010; Roberts et al., 2014
*Hedonic motivations—*enjoyment, flow, playfulness, passion for the task, escapism, desire for better products.	Nambisan, 2002; Nambisan and Baron, 2009; Chen et al., 2012; Roberts et al., 2014
*Altruistic desire to contribute*—expressions of personal values, ideological beliefs, or deeply felt needs	Kollock, 1999; Zeityln, 2003; Zwass, 2010; Roberts et al., 2014

**Table 3:** **Integrative revision of classical motivational taxonomies and co-creation motivators**.

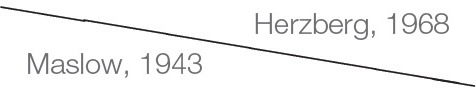	**Extrinsic motivation**	**Intrinsic motivation**
Higher-order needs	Social capital, personal relationships, and identity construction Social standing and renown	Self-esteem, self-efficacy, and self-expression
Lower-order needs	Financial rewards Career advancement	Hedonic motivations Learning

*Source: Based on Guillén et al. (2014)*.

In Table 3 we connect Maslow's (1943) and Herzberg's (1968) classical taxonomies of human motives, in line with Guillén et al. (2014), and our deep review of literature on consumers' motivations to co-create. That is, in the columns of Table 3 we document extrinsic and intrinsic motivations, according to Herzberg's (1968) distinction, and in the rows, we present higher- and lower-order needs, according to Maslow (1943). On the one hand, intrinsic motivations relate to the nature of the activity itself and are rooted in the personal satisfaction that can be achieved by performing the activity (Kozinets, 2002), but extrinsic motivations are utilitarian in nature and associated with attaining external, functional, and practical incentives, distinct from the activity *per se* (Daugherty et al., 2008). On the other hand, lower-order needs are associated with preserving physiological, subsistence (i.e., food, water), safety, and security (i.e., safety, security) needs, whereas higher-order needs have to do with social activities (i.e., love, esteem) and meeting self-actualization aspirations. When consumers who participate in co-creation activities are motivated extrinsically to meet lower-order needs, practical purposes are their real motives (e.g., *financial rewards, career advancement*; see Table 3). When they are motivated intrinsically and seek to meet lower-order needs, they really participate in co-creation activities for practical purposes related to *learning* and enjoying the *personal hedonism* they derive from co-creating new and unique goods. With regard to higher-order needs, consumers often focus on relatedness and likely participate in co-creation activities for extrinsic motives (e.g., *access to social capital, personal relationships, identify with co-creating communities and projects, gain social standing, and renown*). Finally, to meet higher-order needs, s*elf-esteem, self-efficacy*, and *self-expression* can prompt intrinsically motivated consumers to participate in co-creation activities. Thus, given that both intrinsic and extrinsic motivational aspects play a role in explaining consumers' willingness to engage in co-creation activities, we propose:

Proposition 2_A_: Consumers engage in co-creation activities to receive external goods, beyond performing the activity itself, reflecting their extrinsic motivation.
Proposition 2_B_: Consumers engage in co-creation activities to receive internal goods related to performing the activity itself, reflecting their intrinsic motivation.

Although this integrative revision of consumers' motivations to co-create is new to extant literature and offers a clearer general understanding of this issue, some necessary elements are still missing. According to Guillén et al. (2014), both types of motivation classifications (i.e., higher–lower and extrinsic–intrinsic) need to expand to include perspectives that reflect the moral content of motivation, as it relates intrinsically to human life. Thus, even the expanded, integrated view in Table 3 is insufficient for explaining consumers' participation in co-creation activities, especially in the new era in which community members freely share innovative ideas and content to facilitate and leverage value co-creation. There must be other motives, beyond those described in Table 3, which can help us understand why the process of participation in co-creation activities has become so prominent. We posit that these motives revolve around ethical and transcendent issues. In particular, modern consumers devote increasingly more emphasis to ethical values in their purchase decisions (e.g., human welfare, social justice, environmental factors; Shaw et al., 2005). Creative people also might participate for spiritual motives rather than to attain material benefits (Kotler et al., 2010), so consumer participation in value co-creation activities likely aims to improve the usefulness, value, and service of the new product to society. Thus, consumers' willingness to participate is based not only on intrinsic or extrinsic motivations but also on transcendent motives, including the benefit their collaborations have for others in wider society.

#### Ethics and transcendence in consumers' motivations to co-create

**Figure 1:**
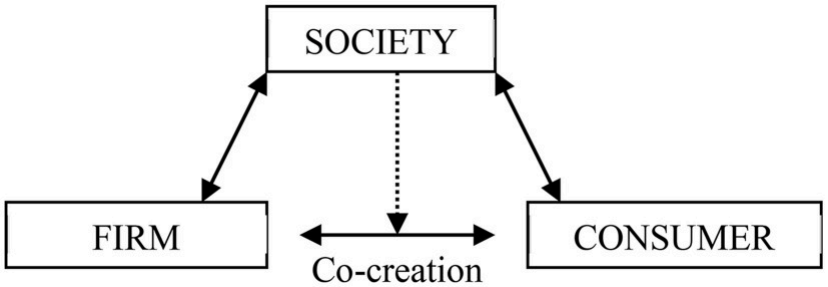
**The S-D Logic: Social values based view of Marketing and Consumers' co-creation activities**.

The *service-dominant (S-D) logic* (Abela and Murphy, 2008) places strong emphasis on *service* and the *co-creation of value* as essential elements for marketing area; it also has had powerful influences on marketing practitioners and researchers (Williams and Aitken, 2011). In this logic, each individual consumer is a resource-integrating, value-creating enterprise (Vargo and Lusch, 2008) that companies must motivate by embedding their business actions in line with the value-laden societal context (see Figure 1). To encourage consumers' participation in co-creation activities, businesses need to behave in accordance with the values that motivate those consumers (Williams and Aitken, 2011). In the modern era, ethics is one such value. That is, in the era of Marketing 3.0., consumers look to products and services not just to meet their needs but also to achieve their spiritual and moral interests and needs (Kotler et al., 2010). Increasingly, consumers are more and more concerned about the effects of their purchase choices, both for themselves and for the world around them (Harrison, 2005). Accordingly, they increasingly look for solutions for their own concerns about how to make the global world a better place, such that they are guided by ethical values in their purchase decisions (Shaw et al., 2005; Hollenbeck and Zinkhan, 2010). For example, the extent to which a product provides freedom of choice, independence, and curiosity are key assessments, and the brand needs to inspire a sense of benevolence, security, equality, environmental friendship, and rules conformity (Shaw et al., 2005).

This ethical axis governing consumers' purchase decisions also offers a proxy for consumers' growing concerns about society's welfare. In modern environments, consumers' decisions to participate in co-creation activities evolve mainly according to their *altruistic desire to contribute* (Kollock, 1999; Zeityln, 2003; Zwass, 2010; Roberts et al., 2014; see Table 1). Thus, transcendent motives based on others' rather than self-interest (Pérez-López, 1998; Guillén et al., 2014) can prompt participation in co-creation activities. As originally proposed by Pérez-López (1998), transcendent motives place concern for others' needs and a sense of service in central positions, prompting people to shift from self-interested to others' perspectives. Consumers guided by these motives, through their actions, likely seek to satisfy others' needs rather than their own, similar to the way that people guided by ethical values such as solidarity, service, or altruism might be inclined to do. Furthermore, transcendently motivated people worry about others' authentic (*moral) human goods* (Melé, 2009: 207), such as *truth, beauty, work, friendship, life*, and *human dignity* (Melé, 2009); have a sense of stewardship; and aim to transcend the individual domain and take the impact of their actions on others, both known and unknown, into account.

According to prior literature, this transcendent dimension, with its close link to ethics, offers a compelling, new, and enriched framework to understand human motivations (Argandoña, 2008; Pastoriza et al., 2008; Melé, 2009; Ferrero and Calderón, 2013; Guillén et al., 2014). As such, and with the recognition that the Marketing 3.0 depends on Web 3.0 platforms and is based on values, it is useful and appropriate to address the motivations of consumers to engage in co-creation activities in this setting. Again following Guillén et al. (2014), we propose expanding the traditional taxonomy of motivations to include this new, third type of motive, beyond intrinsic and extrinsic ones. As we show in Table 4, Herzberg's (1968) motivational framework now is enriched with the incorporation of motivations specifically oriented toward being concerned about others' welfare, in the form of transcendent motives.

**Table 4:** **Extended integrative revision of classical motivational taxonomies and co-creation motivators**.

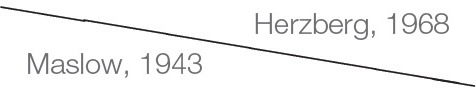	**Extrinsic motivation**	**Intrinsic motivation**	**Transcendent motivation**
Higher-order needs	Social capital, personal relationships, and identity construction Social standing and renown	Self-esteem, self-efficacy, and self-expression	Society's welfare Contribution to the common good
Lower-order needs	Financial rewards Career advancement	Hedonic motivations Learning	Service, help, and collaboration

*Source: Based on Guillén et al. (2014)*.

Applied to a consumer context, and specifically to engagement in co-creation activities, a transcendent motivation might mean practices such as *collaboration, cooperation, help, and service*, in an effort to meet lower-order needs. Consumers might engage in these practices during the realization of co-creation activities for practical reasons related to their needs to grant knowledge, experience, skills, or competencies that they have acquired and believe might be useful to others. Consumers also might cooperate in the hope they can receive help when they are in need. However, when consumers engage in co-creation activities to meet their higher-order needs, the collaboration is usually regarded as an end in itself. The consumers think authentically about *transcending their personal sphere to acknowledge the welfare of wider society* and *contribute to the common good*. In contributing to the common good, consumers seek to bring about a society that features, for example, unpolluted air, social cohesion, educational goods, environmentally friendly products and services, and healthy and practical offerings (Melé, 2009). Thus, consumers can fulfill their spiritual needs and develop their human side when participating in co-creation activities, which constitutes an increasingly critical demand among creative consumers today (Kotler et al., 2010).

Table 4 thus synthesizes the several motives that consumers exhibit when they decide to collaborate and engage in co-creation activities. In this new era, consumers increasingly emphasize ethical values and seek to make purchase decisions in a conscious manner, such that they think carefully about the environmental, ethical, and social costs (Beagan et al., 2010). Our extended taxonomy, following Guillén et al. (2014), incorporates a transcendent motivation dimension and reveals various motives arising on the scene that influence consumers' intentions to participate in co-creation activities, with special attention to ethical, transcendent ones. Although the influences of these motivations vary in strength, they are complementary, supplementary, and potentially simultaneous in both time and action. In the modern era of ethical consumption (Harrison, 2005; Shaw et al., 2005; Beagan et al., 2010) and Marketing 3.0 (Kotler et al., 2010), the transcendent motives occupy perhaps the most critical role in encouraging consumers to participate in co-creation activities. Consistently, we propose:

Proposition 3: The ethical values–driven Marketing 3.0 era boosts consumers' transcendent motivations in relationships and decision making about brands and companies (e.g., purchases, collaboration).
Proposition 4: Consumers engage in co-creation activities to meet their need to give to others, care for others' welfare, reflecting their transcendent motivation.

### Consumer co-creation processes

#### Direct effects on consumers and companies

In seeking new ways to create consumer value, current marketing developments, such as the S-D logic, may prove especially useful. The S-D logic is based on the premise that companies do not deliver value but rather work out value proposals. Consumers themselves individually create value by using or consuming products and services. This new approach also emphasizes that the customer's participation in the product and service experience is indispensable for creating more value, such that both consumers and company employees are active participants in the creative process. Two further elements are implicit to this process and should be fostered by companies: consumer empowerment and consumer engagement. Both elements have been addressed repeatedly in co-creation literature as essential to allow for the process to flow and generate positive outcomes for both consumers and companies.

*Consumer empowerment* implies that the company delegates power to consumers to co-create new products and services (Zimmerman and Warschausky, 1998; Cova and Pace, 2006; Füller et al., 2009). This delegation is increasingly possible in the new era; due to new technologies, consumers have been enabled to interact with the world on various levels (e.g., personal, dyad, group, community), as well as observe and experience distant things as if they were real (Kozinets et al., 2004). The Internet and new Web 3.0 platforms provide consumers with an accessible medium to express their opinions and observations about purchase decisions and product/service characteristics; to share knowledge with companies; and to engage in new product or services designs, in meaningful, challenging new product development tasks or other product-related activities (Füller et al., 2009).

Accordingly, consumer empowerment strengthens consumers' perceptions of their own self-determination and self-efficacy (Füller et al., 2009). Thanks to advances in new technologies (Web 3.0; Cova and Pace, 2006), consumers increasingly combine their resources and skills with others' resources to create virtual spaces in markets, where they exert powerful influence, establish their credibility, and can develop their own identity (Cova and Dalli, 2009; Füller et al., 2009). The Internet and new *ICTs* also allow consumers to interact with others, role play, test their social skills (which strengthens their sense of self-identity), and enjoy mastery experiences (which increases self-efficacy perceptions) (Hamburger et al., 2008; Füller et al., 2009). In turn, other positive outcomes for consumers and companies are likely. First, in terms of the effects for companies, consumer empowerment may cause consumers to perceive that the brand that has assigned them more power produces higher quality products and services, leaving them more motivated and committed to co-creation activities (Zeithaml and Bitner, 2003), as well as the related brands and companies. Second, regarding the effects on consumers, the perceived quality of consumers' own contributions to the co-creation process should enhance their satisfaction, with both their own services and the tasks (Kelley et al., 1990; Vega-Vazquez et al., 2013). When these positive emotional experiences occur repeatedly, consumers' loyalty to the focal brands and companies gets reinforced, such that a virtuous cycle initiates (Lam et al., 2004).

*Consumer engagement* is also essential to co-creation processes, and those processes may vary depending on its level (DeFillippi and Roser, 2014). Patterson et al. (2006) define *consumer engagement* as the level of physical, cognitive, and emotional presence the consumer devotes to a relationship with an organization, involving vigor, dedication, absorption, and interaction. Such traits are fostered by Web 3.0 platforms that enable consumers to share, socialize, learn, advocate, and co-develop (Brodie et al., 2013). As a result, high-quality relationships (consumer-to-consumer and consumer-to-business) arise from continuous dialogue (Prahalad and Ramaswamy, 2004; Jaworski and Kohli, 2006; Auh et al., 2007), with important and valuable outcomes for both consumers and companies.

Specifically, through the *social relationships* established in the co-creation process, consumers engage easily in dialogue with others in each stage of the product design or delivery process (Payne et al., 2008), which induces *consumer learning*. This dialogue during the co-creation process encourages shared emotions, behaviors, and knowledge (Payne et al., 2008), resulting in interactive processes of learning (Ballantyne, 2004). Through virtual experiential interactions and encounters, consumers perceive that their engagement in the co-creation activities ensures the utilization of their own personal resources (Payne et al., 2008), while also helping them improve and reach higher levels of learning and knowledge, because with others, they have the opportunity to create value through customized and co-produced offerings. Co-creation processes enable them to communicate directly with one another and share their experiences, which also can lead to *personalized interactions*, depending on how each consumer prefers to interact with the company (Prahalad and Ramaswamy, 2004). As a result, consumers' co-creation activities likely boost *consumer satisfaction* with the co-creation process and their maintained social relationships (Bowden, 2009), which then becomes the seed of social capital within the social community (e.g., Nuttavuthisit, 2010; Linuesa-Langreo et al., 2015) and ultimately should boost consumers' *creative thinking* (e.g., Füller et al., 2009; Ramaswamy and Gouillart, 2010).

With regard to the benefits for companies, several studies show that consumer co-creation processes increase *consumer trust* in the community setting in which their social relationships have developed (e.g., Casalo et al., 2007; Füller et al., 2009; Hollebeek, 2011; Brodie et al., 2013), as well as *consumer loyalty* (e.g., Andersen, 2005; Casalo et al., 2007; Schouten et al., 2007) and *commitment* to the social community (e.g., Chan and Li, 2010). The involvement of these external agents in the co-creation process, who work as partial employees (Dong et al., 2008), leads to new product ideas that can satisfy new and emerging needs, as well as lowered product development, design, and marketing costs (Ramaswamy and Gouillart, 2010). Because consumers prefer not to have products and processes imposed on them, co-creation processes help differentiate the company from competitors, because they enable consumers to co-construct to suit their own contexts and needs (Prahalad and Ramaswamy, 2004). The important benefits from encouraging consumers to engage in co-creation processes thus entail, for example, *cost effectiveness, risk reduction* (Prahalad and Ramaswamy, 2004), and *differentiation* (Ramaswamy and Gouillart, 2010).

Of all the benefits for companies though, *consumer brand loyalty* is perhaps the most well documented one, and are defined as a “deeply held commitment to re-buy or re-patronize a product/service consistently in the future” (Oliver, 1999: 34), Brands usually evoke emotional and symbolic issues (Aaker, 1999), including two basic elements—authenticity and sincerity—that largely define the image that consumers develop about a specific company (Keller, 1993; Kaplan and Haenlein, 2010) and the affect and loyalty they devote to a brand (Aaker, 1999). Brand perceptions result from consumers' relationships with brands, and these relationships mimic interpersonal relationships (Fournier, 1998), so satisfactory co-creation processes logically should cause perceptions of brand authenticity and sincerity. Co-creation actively seeks to facilitate interactions between consumers and companies, so creating and maintaining an authentic, open dialogue and incorporating consumer needs in new product or service development processes should enhance brand authenticity perceptions. If the co-creation process also gets communicated to the general target group with a sincere storyline, brand sincerity perceptions should grow stronger (Dijk et al., 2014). Co-creation creates such close consumer–brand interrelationships that consumers' *brand loyalty* is increasingly probable in these settings (Luo et al., 2015).

In summary, important benefits derive from new business insights into co-creation, for both consumers and companies. Table 5 summarizes these benefits, revealing the diverse, complex value that consumer co-creation processes provide. On these theoretical grounds and arguments, we propose:

Proposition 5: When consumers engage in co-creation activities, it boosts value for both consumers and companies, in multiple forms.

**Table 5:** **Positive effects of co-creation**.

**Value for the consumer**	**Authors**
Consumer empowerment, self-determination, and self-efficacy	Zimmerman and Warschausky, 1998; Cova and Pace, 2006; Cova and Dalli, 2009; Füller et al., 2009
Consumer engagement, access to social inter-relationships	Payne et al., 2008; Brodie et al., 2013; Jaakkola and Alexander, 2014
Consumer satisfaction	Grönroos, 2008; Bowden, 2009; Vega-Vazquez et al., 2013
Consumer learning	Payne et al., 2008
Creative thinking	Füller et al., 2009; Ramaswamy and Gouillart, 2010
Personalized co-creation experiences	Prahalad and Ramaswamy, 2004
**Value for the Company**	**Authors**
Consumer trust	Casalo et al., 2007; Hollebeek, 2011; Brodie et al., 2013
Consumer commitment	Chan and Li, 2010
Consumer loyalty	Andersen, 2005; Auh et al., 2007; Casalo et al., 2007; Schouten et al., 2007
Cost effectiveness and risk reduction	Ramaswamy and Gouillart, 2010
Differentiation	Prahalad and Ramaswamy, 2004
Consumer brand loyalty	Kim and Slotegraaf, 2015; Luo et al., 2015

#### Co-creation of ethical products: Effects on consumers and companies

Consumers are increasingly willing to integrate ethics into their product purchase decisions. By “ethical” products, we mean products that reflect one or several social, moral, or environmental principles that could influence purchase decisions. A product cannot be ethical *per se*, but it can be augmented by ethical considerations or attributes that are perceived positively (Crane, 2001). Thus, because ethical consumers actively “adhere” to social and environmental principles (Strong, 1996), the presence of ethical characteristics in the product might enhance consumer engagement in co-creation processes today, in the new ethical valued-driven Marketing 3.0 era. The more an object relates to these (ethical) values, the more involving it may be for consumers (e.g., O'Cass, 2001).

As noted previously, consumers' co-creation offers important benefits for both consumers and companies; we also posit that these benefits increase when the product or service they are co-creating features ethical characteristics. For example, at the company level, emerging research on brand building processes suggests that consumers' trust in brands depends on whether they perceive that brands are ethical or, more important, offer products and services that are just, honest, and trustworthy (Singh et al., 2012). Considering that altruistic and courteous behaviors prompt enhanced positive affect (Sung and Kim, 2010), a brand that is perceived as ethical will elicit positive emotional responses among its consumers and invoke a stronger level of brand affect among them (Glomb et al., 2011). At the consumer level, when co-creation processes involve ethical products and services, consumers gain more value from their participation. In parallel with findings that reveal that committing moral deeds creates a sense of purpose, meaning in life, and relative gains in happiness (Hoffman et al., 2014), consumers who co-create ethical products and services likely experience good feelings and increased consumer satisfaction, as well as better, more personalized co-creation experiences. Also, research reveals that long-term, supportive collaborations are likely to be enhanced when the decisions that each party to the relationship makes evoke perceptions of fairness or ethicality (Ruiz-Palomino et al., 2013).

*Brand loyalty* tends to apply to ethical companies because it is based on a consumer commitment for future transactions. Thus, when consumers perceive fairness in a company's service or product transaction, as well as in the process for handling customer claims, repurchase intentions grow (Hellier et al., 2003), which in turn may increase loyalty to the company. Customer–brand relationships cannot be sustained in the face of ethical misconduct by the company (Roman, 2003; Huber et al., 2010). In view of these arguments, the consumer co-creation process for developing ethical products should lead to increased positive benefits for both consumers and companies. Thus we propose:

Proposition 6: The value of consumers' engagement in co-creation activities for both companies and consumers is higher when co-creation involves ethical products.

## Integrative model of the co-creation process, its causes and its effects

**Figure 2:**
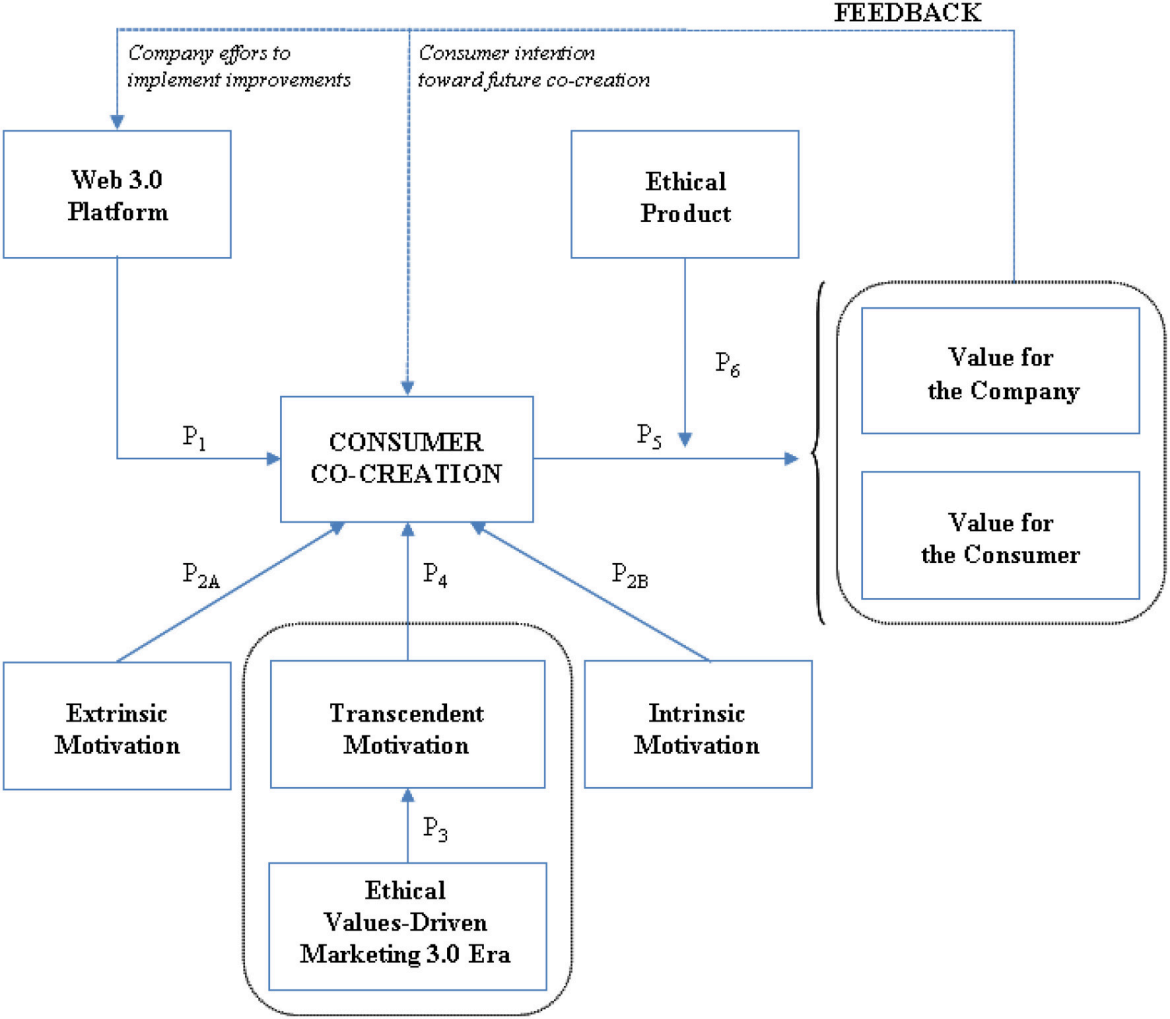
**An integrative model of the causes of the co-creation process and its positive effects**.

Reflecting our integrative literature review and the resulting theoretical propositions, we developed the integrative model of the causes and effects of consumers' engagement in co-creation activities in Figure 2. This model incorporates both theoretical and empirical contributions from prior literature and seeks to affirm a better understanding of the process of consumer co-creation, both is antecedents and consequences. Furthermore, it includes ethics as an essential element. As described in the previous sections, several areas of research provide the foundation for this proposed model, including consumer co-creation (e.g., Prahalad and Ramaswamy, 2004), Web 3.0 platforms (e.g., Kalaignanam and Varadarajan, 2006), motivation (e.g., Maslow, 1943; Herzberg, 1968; Deci and Ryan, 1985; Pérez-López, 1998), ethical product characteristics (e.g., Crane, 2001), and the positive effects of co-creation on both consumers and companies (e.g., Prahalad and Ramaswamy, 2004; Füller et al., 2009; Ramaswamy and Gouillart, 2010; Brodie et al., 2013; Luo et al., 2015).

The first part of the model depicts the essential elements that initiate and influence the co-creation process. In particular, the development of the Internet and *ICTs* allows companies to provide Web 3.0 platforms and helps consumers interact widely and easily with both other agents and the company to co-create new products and services (P_1_ in Figure 2). These new interactive spaces also support strong relationships and a sense of social community, through the easier, increased interactions with others, learning processes, and social links. This model also includes motivational factors that might lead consumers to engage in co-creation activities. Extrinsic motivations (financial rewards, personal relationships, identity construction, social standing, and renown) and intrinsic motivations (hedonic factors, learning, self-esteem, self-efficacy, self-expression) are well-known from prior literature (P_2A_ and P_2B_ in Figure 2). However, in modern society, with its focus on ethical and social values, consumers seek out companies that offer products and services to address social, economic, and environmental problems (Kotler et al., 2010). Creative people also prefer to follow spiritual, social, or ethical motives, rather than simply attaining material or personal goals (Kotler et al., 2010). In this sense, consumer participation in value co-creation activities is more likely to reflect transcendent motivations to improve the usefulness of products or services and offer value to society (P_4_ in Figure 2). In a values-focused era, ethical values provide guides for consumers, and ethical values, such as sharing knowledge, experience, skills, or competencies or contributing to the common good, can underlie the transcendent motivations of consumers to engage in co-creation activities (P_3_ in Figure 2).

The second part of the model highlights the positive effects of co-creation processes on both consumers and companies (P_5_ in Figure 2). Value co-creation thus is based on interactive, social processes promoted by consumers and companies, in which valuable resources are integrated and value is distributed among agents (Prahalad and Ramaswamy, 2004). Thus, consumers work as partial employees who adhere voluntarily to the inspiring community project, promoted by companies. Two core outcomes for consumers derive clearly from these processes: *consumer empowerment*, spanning self-determination and self-efficacy perceptions, and *consumer engagement*, which produces access to social relationships, creates effects that are positive in and of themselves (i.e., consumer satisfaction with the service, consumer learning, personalized experiences of co-creation), and sparks synergic effects and benefits for the social communities and companies involved (trust, commitment, loyalty). The model also reveals that companies benefit from cost effectiveness (consumers as partial employees), risk reduction (Ramaswamy and Gouillart, 2010), market differentiation (Prahalad and Ramaswamy, 2004), and brand loyalty (Kim and Slotegraaf, 2015; Luo et al., 2015). Finally, the entrance of ethical products on the scene of co-creation processes causes the previously described positive effects, for both consumers and companies, to increase substantially (O'Cass, 2001; P_6_ in Figure 2).

The model highlights the important value generated by consumers and companies. Companies should leverage the benefits of fostering consumer empowerment and engagement in co-creation processes. Because we increasingly are moving toward a world in which value results from an implicit negotiation between the individual consumer and the company (Prahalad and Ramaswamy, 2004), the co-creation of value with consumers is a new business model that companies should embrace, so that they can compete in an efficient, differentiated manner (Prahalad and Ramaswamy, 2004; Ramaswamy and Gouillart, 2010). Although continuing improvements to Web 3.0 platforms will help feed the consumer co-creation process (dashed lines in Figure 2), they are useless without consumers' intentions to engage in future co-creation activities. Such intentions can be fostered if consumers perceive that they will gain multiple forms of benefits from engaging in co-creation activities, as well as insofar as they participate more in such processes, which should give them confidence in their ability to complete tasks and participate in value co-creation, lead them to take ownership of the activities, reduce their risk perceptions, and allow them to enjoy the whole experience more (Dong et al., 2008). A feedback process that indicates how, *ceteris paribus*, increased consumer participation in co-creation causes the process to continue is also represented in the model (dashed lines in Figure 2).

## Conclusions

Our research represents a relevant contribution to the fields of business and marketing, specifically to the area of consumers' co-creation value. Our first contribution is the valuable synthesis of representative literature and expanded, diversified knowledge that we have gained around the co-creation process involving consumers. Through an integrative literature review methodology (Torraco, 2005; Yorks, 2008), we have developed a conceptual model to describe both the antecedents and the positive outcomes of consumers' engagement in co-creation processes. Based on ethical theory, we have provided a fresh new understanding by offering sound support to the role that ethical values and transcendent motives play in boosting consumers' co-creation processes, which fills a missing gap in afore-mentioned literature review.

The second important theoretical contribution refers to the advances that our article represents for the better understanding of the reasons why consumers engage in value co-creation activities with companies. Specifically, our study incorporates transcendent motives as a complement and extension to the classical intrinsic–extrinsic motivation theory (Herzberg, 1968) to understand this process. Although abundant literature emphasizes intrinsic (i.e., hedonic motivations) and extrinsic needs (i.e., financial rewards) as responsible for consumers' engagement in co-creation activities, our literature review elucidates other important, transcendent needs in this area. Consumers' willingness to participate is based not only on intrinsic or extrinsic motives but also on the need to benefit, through their collaborations, third parties in the wider society, which corresponds to the new ethical values-driven Marketing 3.0 era.

Our third contribution refers to the adequate specification that our model makes about the influential roles of new technologies—web 3.0 platforms—and the Marketing 3.0 paradigm to better understand the process leading consumers to engage in value co-creation processes. In these new contemporary times, consumers increasingly are emphasizing ethical values and seeking to make purchase decisions in a conscious manner, by carefully thinking about the ethical, environmental, and social costs derived. Accordingly, the way consumers are conceived and seen must be changed in order to adapt to the new times successfully. Consequently, the ethical values-driven Marketing 3.0 paradigm, which has recently emerged, occupies an essential role to understand the process of consumers' engagement in value co-creation processes. Under the umbrella of this paradigm, companies are seen as entities that must treat consumers as human beings, with intelligence, heart, soul, and spirit, as well as aspire to live such ethical values as cooperation, friendship, and human welfare. Therefore, as a result of consumers perceiving this congruence in ethical values with companies, the co-creation process is helped to start and develop over time, which is clearly identified in our conceptual model. Of course, to implement and follow the Marketing 3.0 paradigm and thus promote collaboration with consumers adequately, companies need the new advances in *ICTs*. Web 3.0 platforms, and its continuous improvements, thus, offer new tools that consumers can use to interact with companies and other agents, as well as incentives for creating new product and services.

Finally, we importantly contribute to literature by identifying the positive outcomes and the process by which these are obtained when consumers' engagement in co-creation activities occurs. In this sense, one important part of our conceptual model refers to the specific positive outcomes that these activities entail for both companies, and importantly, consumers, which aims to fill a void in literature. Our integrative review of literature revealed that these activities might result in important, very valuable outcomes for companies (i.e., consumer loyalty) and consumers (e.g., consumer satisfaction), but depend on whether the co-created products or services feature ethical characteristics. The presence of ethical characteristics in the product or service co-created is sensitive so as to strengthen the value created for both consumers and companies. Because one important motivation for co-creation is the value which is created for others, when co-creation processes involve ethical products and services, consumers tend to gain more value from their participation. In parallel with findings that reveal that committing moral deeds creates a sense of purpose, meaning in life, and relative gains in happiness, consumers who co-create ethical items are more likely to experience good feelings and satisfaction as well as better personalized co-creation experiences. Also, these consumers are expected to increase their trust, commitment and loyalty to the brand and the company with which they are collaborating. All these positive outcomes would also be fostered insofar consumers perceive they gain multiple forms of benefits from engaging in co-creation activities, as well as they will participate more and more in such processes.

All in all, our integrative review of literature offers new understandings around the engagement process of consumers in co-creation activities, and has given rise to some interesting conclusions that should be well considered in business and marketing management. Companies must collaborate with consumers, who play a key role in generating value and competitive advantages by providing information, fresh ideas, and co-creating new, improved products and services. They are sources of creativity as well as sources of social, ethical values imprints in the product design, and development processes, which is imperative to be successful in the new contemporary times. The consumer role has evolved so much that today consumers are now described as active agents, protagonists, or value co-creators. These roles also converge to describe not just now actively and constructively consumers are today but also the importance of their market experiences, joint activities, and relationships with companies. That's why managers should acquire an integral understanding of the antecedents making consumers engage in value co-creation activities. With this in mind, our integrative review allows us to highlight the important role played by that instrumental devices (i.e., Web 3.0 platforms) and personal mechanisms (i.e., intrinsic, extrinsic motives) in fostering these activities. However, new to literature, special emphasis has been laid on ethical values and, specifically, on personal transcendent motives as antecedents. Modern society is increasingly focusing on ethical values, along with consumers seeking companies that offer creative products, services that truly solve the current social, economic, and environmental problems. Humanity, morality, and spirituality are common elements behind these creative solutions (Zohar, 1990). In this vein, to encourage consumers' engagement in co-creation activities and creativity, companies' alignment with ethical and transcendent values should not be obviated, nor the design and development of ethical, responsible items.

## Implications for research and practice

For academic researchers, our theoretical model and integrative literature review should serve as stimulants of further studies on the topic, in that they provide a reference or starting point for additional research. Our findings demonstrate the need for understanding the causes and effects of consumers' participation in co-creation processes, as well as the positive effects for both consumers and companies. The findings further suggest that when consumers feel empowered, with passion and a sense of ownership, they are willing to contribute extensively for the benefit of the company. Thus, companies need to learn about their consumers' desires and needs, beyond normal exchange processes, especially considering the importance of ethical and social values for consumers today.

For managerial practice, various implications emerge from this study. The first one is related with the potential value which consumers who are willing to participate in value co-creation activities. Managers should assess the potential value of their communities of proactive customers for a greater innovation, brand loyalty, differentiation, and augmentation of competitive advantage to their companies. Consequently, organizations should take a long-term view of their customer relationships, rather than a short-term financial perspective. Within a short-term approach, companies are unlikely to boost consumers' co-creation participation; consumers need to perceive their relationship with the company as equitable, which takes time. A long-term perspective is also more suitable, considering consumers' increasing interests in ethical and social values. It might ensure that consumers' transcendent motives align with companies' interests to contribute, ultimately, to the general welfare of society.

One second implication is related to the necessary commitment of the top management team with favoring these co-creation processes, and thus allow for these processes to develop optimally. Managers who seek new ways to involve consumers in co-creation activities should institute cultural changes in their organizations. Co-creation initiatives require flexible organizational structures, oriented toward engaging in frequent contacts with consumers and meeting their needs. This view is important to build emotional bonds between consumers and companies as well as to encourage consumers' engagement in co-creation activities (Grayson, 1998). Accordingly, managers should take this commitment in mind when planning, organizing, leading, and monitoring the activities of their employees.

One third implication is the creation and maintenance of relevant communication channels with consumers through platforms such as Web 3.0. These efforts can translate into enhanced encounters, supporting cognition, emotion, and action-based learning for both consumers and companies, and thus result in a proactive community that fosters consumers' loyalty to the community and to the company. Although consumers' engagement in value co-creation activities strengthen these ties, and might enhance the level of community commitment on itself, the more companies invest and develop efforts in connecting consumers, the better to retain on loyal both to the community and the company. The design of good interaction channels with consumers is thus an important element to implement the value co-creation strategy, and thus make the creative process initiated with consumers work properly.

Finally, companies should be cautious about the negative potential consequences of empty value co-creation strategies. Due to the low cost and popularity of social media, many companies almost blindly take for granted advantages to initiate various value co-creation activities. However, these activities are not just about interacting with customers and managers; they rather require a strategy based on careful planning and implementation through Web 3.0 tools. Thus, managers should create policies based on ethical principles and the Marketing 3.0 paradigm to obtain a long term and effective business development. Business brands should behave ethically regardless of the potential impact on the bottom line. However, in a highly interconnected world that has made brands more transparent, truly ethical behavior of companies and customers based on transcendent motives will be necessary to succeed in any marketplace.

## Limitations and further research

Despite these contributions, we acknowledge some limitations and accordingly propose new avenues for research. First, empirical data are needed to validate the conceptual model and theoretical propositions. In particular, Tables 2–5 synthesize findings and suggestions from prior literature; those summaries should be tested empirically in the particular setting of consumers who are participating in co-creation activities. Other moderating effects also can be examined, such as social justice perceptions or relevant contextual variables. For example, studies might analyze the effectiveness of co-creation processes when consumers perceive a balance in their relationship with the company, or else document any negative effects that arise when a consumer perceives an unbalanced relationship. Second, we focused on business-to-consumer interactions, but other beneficial relationships may also tend to arise, whether business-to-business links or relationships with government or third-party agencies. Additional research focused on understanding the co-creation processes that arise from these relationships thus could helpfully nourish from integrating the perspectives addressed here. Third, in line with our research goals, we concentrated on the positive effects of consumers' engagement in co-creation processes. However, researchers could address the specific variables that might produce negative aspects, such as negative consumer sentiments, bad brand experiences, or trust reduction. Finally, a longitudinal study would offer a more dynamic view of value co-creation and help determine how these effects evolve over time.

## Author contributions

All authors listed, have made substantial, direct and intellectual contribution to the work, and approved it for publication.

## Funding

This work was funded by The Ministry of Economy and Competitivity (Spain), Research Project reference ECO2014-59688-R, Programa Estatal de Investigación, Desarrollo e Innovación Orientada a los Retos de la Sociedad, Plan Estatal de Investigación Científica y Técnica y de Innovación 2013–2016.

### Conflict of interest statement

The authors declare that the research was conducted in the absence of any commercial or financial relationships that could be construed as a potential conflict of interest.
